# Adipose-Derived Mesenchymal Stem Cells Improve Acute Liver Injury: A Mechanistic Study Based on the TLR4/MyD88/NF-κB Pathway

**DOI:** 10.3390/ijms262411798

**Published:** 2025-12-06

**Authors:** Zhongfa Wang, Minjuan Li, Xingxing Yan, Yanchen Liu, Pengkun Yang, Wenzhong Liu, Weijun Guan

**Affiliations:** 1 Department of Animal Genetic Resources, Institute of Animal Science, Chinese Academy of Agricultural Sciences, Beijing 100193, China; 19722725957@163.com (Z.W.); 17806280540@163.com (M.L.);; 2 Department of Animal Genetics, Breeding and Reproduction, College of Animal Science, Shanxi Agricultural University, Jinzhong 030600, China

**Keywords:** Luopan Mountain pig, ADSCs, TLR4/MyD88/NF-κB, liver regeneration, anti-inflammatory, antioxidant

## Abstract

Acute liver injury (ALI) involves complex pathogenesis and lacks effective clinical therapies. Although mesenchymal stem cells (MSCs) demonstrate therapeutic potential, the role and mechanisms of adipose-derived mesenchymal stem cells (ADSCs) from Luopan Mountain pigs remain unclear. This study assessed the therapeutic potential of Luopan Mountain pig ADSCs in a D-GalN-induced rat model of ALI and investigated its association with the TLR4/MyD88/NF-κB axis. Results showed that ADSCs transplantation significantly improved liver function (by reducing ALT, AST, and TBIL levels and increasing ALB levels) and alleviated histopathological damage in liver tissue. Mechanistically, ADSCs conferred multi-faceted hepatoprotection via inhibition of the TLR4/MyD88/NF-κB axis, synergistically downregulating proinflammatory factors (TNF-α, IL-1β, IL-6, IL-8), enhancing antioxidant enzyme activity (SOD, GSH-PX), and promoting the expression of the hepatocyte regeneration marker Ki67. We demonstrate for the first time that Luopan Mountain pig ADSCs synergistically repair acute liver injury by inhibiting the TLR4/MyD88/NF-κB pathway, offering novel insights for cell therapy in ALI.

## 1. Introduction

ALI is an acute inflammatory response in the liver triggered by multiple etiologies, often accompanied by hepatocyte necrosis and abnormal liver function [1]. Its pathogenesis is complex, involving multiple aspects such as oxidative stress [2], inflammatory response [3], and immune regulation [4], with oxidative stress playing a pivotal role [5]. Iron overload leads to reactive oxygen species (ROS) accumulation, thereby inducing hepatocyte injury [6]. Studies confirm that iron metabolism disorders are closely related to the progression of acute liver injury. During the injury process, the expression of iron regulatory proteins such as transferrin receptor 1 and ferritin light chain is significantly upregulated [7]. Furthermore, acute liver injury is often accompanied by intense inflammatory responses. Platelets interact with Kupffer cells via the CLEC-2 receptor [8], promoting TNF-α production and exacerbating neutrophil infiltration and liver injury [9]. Multiple signaling pathways participate in regulating the development of acute liver injury, including the PI3K/AKT [10], AMPK [11], and NF-κB pathways [12]. Research indicates that nanoparticles based on plant polyphenols like usnic acid can alleviate oxidative stress and inflammatory responses by modulating the Nrf2/HO-1 signaling pathway [4]. Meanwhile, lycopene mitigates polyhalogenated carbazole (PHCZ)-induced liver injury by inhibiting ROS/PI3K-AKT/NF-κB axis [13].

In recent years, treatment strategies for ALI have continuously emerged, including pharmacotherapy [14,15], nanotechnology [16,17], and cell therapy [18]. Among these, mesenchymal stem cells (MSCs), particularly ADSCs, have demonstrated significant potential due to their potent immunomodulatory, antioxidant, and tissue regeneration capabilities [19,20]. Studies indicate that ADSCs secrete anti-inflammatory factors, suppress proinflammatory factor expression, and upregulate antioxidant enzymes (e.g., SOD, GPx), thereby reducing oxidative stress and protecting hepatocytes [21,22]. Furthermore, ADSCs stimulate hepatocyte proliferation and angiogenesis by secreting HGF and VEGF [23], accelerating liver function recovery [24]. By inhibiting the TGF-β1/Smad signaling pathway, ADSCs also reduce collagen deposition and improve liver fibrosis [25]. In a CCl_4_-induced canine acute liver injury model, ADSCs significantly lowered serum AST and ALT levels while restoring hepatic glycogen synthesis capacity [26].

D-GalN is a widely used hepatotoxic agent that mimics the pathological process of acute liver injury by inducing oxidative stress and inflammatory responses [27]. Its core mechanism involves the depletion of uridine triphosphate (UTP) during D-GalN metabolism within hepatocytes, which subsequently inhibits RNA and protein synthesis, ultimately leading to hepatocyte necrosis [28,29]. Crucially, D-GalN’s proinflammatory effects primarily depend on endogenous damage-associated molecular patterns (DAMPs) released following hepatocyte necrosis [30]. Among these, high-mobility group box 1 (HMGB1), a key DAMP, is released extracellularly and recognized by Toll-like receptor 4 (TLR4) [31,32]. TLR4, a core pattern recognition receptor in the innate immune system [33], activates the NF-κB signaling pathway upon binding HMGB1 by recruiting myeloid differentiation factor 88 (MyD88), thereby driving inflammation and establishing a “necrosis–inflammation” positive feedback loop [34,35]. Thus, excessive activation of the TLR4/MyD88/NF-κB pathway serves as a critical bridge linking D-GalN-induced initial liver injury to subsequent inflammatory responses.

Despite the significant potential of ADSCs in ALI treatment, their specific mechanisms of action remain incompletely elucidated [36]. There are also differences in the therapeutic effects of ADSCs from different species on ALI [37]. As a distinctive local pig breed in China, Luopan Mountain pigs are renowned for their robust disease resistance and unique metabolic characteristics. These unique genetic and phenotypic traits may be reflected in the functional activity of their ADSCs [38], yet research on their ADSCs remains unexplored. Given the high physiological and metabolic similarity between pigs and humans [39], this study selected Luopan Mountain pig ADSCs to systematically evaluate their therapeutic efficacy against D-GalN-induced rat ALI. We further investigated whether their mechanism of action involves regulating the TLR4/MyD88/NF-κB signaling pathway, aiming to provide new perspectives and experimental evidence for cell therapy strategies based on unique genetic resources.

## 2. Results

### 2.1. Characterization of ADSCs from Luopan Mountain Pigs

**Figure 1:**
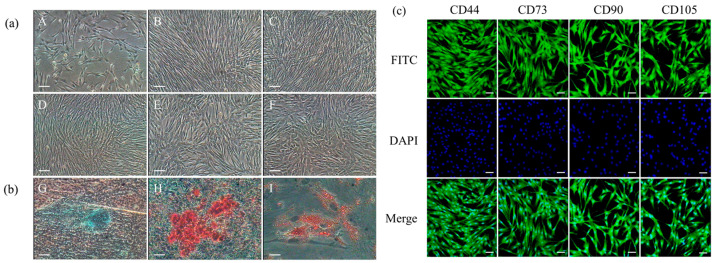
Biological characterization of ADSCs. (**a**) Cell morphology of ADSCs: P0 (A), P5 (B), P10 (C), P15 (D), P20 (E), and P25 (F) (scale bar = 100 μm). (**b**) Differentiation potential assessment. Chondrogenic (G) (scale bar = 100 μm), osteogenic (H) (scale bar = 100 μm), and adipogenic (I) induction (scale bar = 20 μm). (**c**) Immunofluorescence staining (scale bar = 20 μm).

ADSCs from the Luopan Mountain pig were isolated and cultured using the tissue block adhesion method. The cells exhibited a typical elongated spindle shape and grew in vortex-like colonies (Figure 1a). Immunofluorescence analysis confirmed positive expression of the MSCs’ surface markers CD44, CD73, CD90, and CD105 (Figure 1c). Furthermore, the cells demonstrated multipotent differentiation capacity: after a 15-day chondrogenic induction, Alcian blue staining showed positive blue nodules; after a 14-day osteogenic induction, carmine staining showed red calcified nodules; and Oil Red O staining revealed red lipid droplets following a 7-day adipogenic induction (Figure 1b). These results confirm that the cultured cells exhibit the biological characteristics of ADSCs.

### 2.2. Detection of Homing Capacity of Liver Tissue-Derived ADSCs

**Figure 2:**
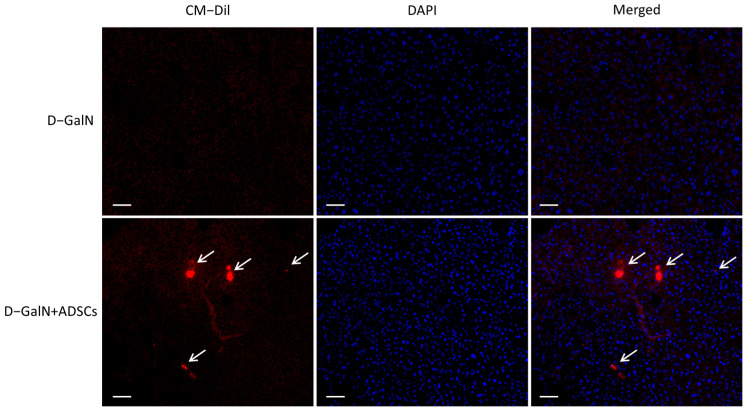
ADSCs in liver tissue. Fluorescent microscopy image of liver tissue 24 h post-transplantation. White arrows indicate CM-Dil-labeled ADSCs (red fluorescence) localized to the injury site. Scale bar = 100 μm.

Fluorescent microscopy revealed a significant accumulation of CM-Dil-positive cells in the liver tissue of the ADSC-transplanted group versus the D-GalN group (Figure 2). This finding indicates that ADSCs transplanted via the tail vein can specifically home to the site of liver injury.

### 2.3. ADSC Transplantation Improved Survival Rates in ALI Rats

**Figure 3:**
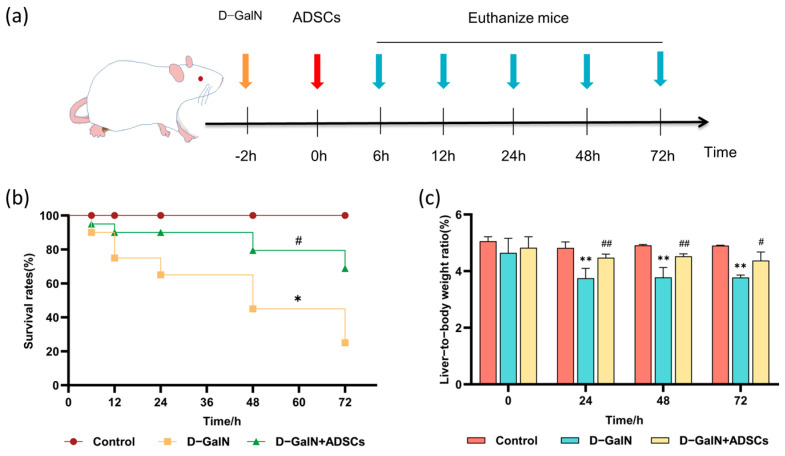
Effects of ADSC transplantation on survival rate and liver weight ratio in ALI Rats. (**a**) Experimental timeline. (**b**) Survival curves for each group (*n* = 15). (**c**) Liver weight ratio (mean ± SD). * *p* < 0.05, ** *p* < 0.01 vs. control group; ^#^ *p* < 0.05, ^##^ *p* < 0.01 vs. D-GalN group.

To evaluate the therapeutic efficacy of ADSC transplantation in ALI rats, we recorded survival rates across all groups (Figure 3a). ADSC transplantation markedly increased the survival rate of ALI rats versus the D-GalN group (*p* < 0.05; Figure 3b) while concurrently enhancing their liver weight coefficient (*p* < 0.05; Figure 3c).

### 2.4. ADSC Transplantation Improves Liver Tissue Injury in ALI Rats

**Figure 4:**
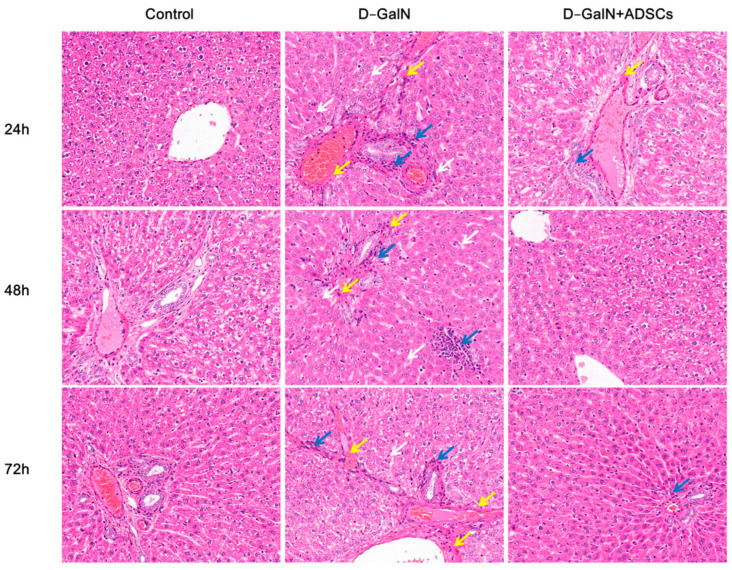
HE staining. White arrow: dilated and congested hepatic sinusoids; blue arrow: inflammatory cell infiltration; yellow arrow: fibrous tissue proliferation. Scale bar = 100 μm. (*n* = 3).

HE staining showed that, relative to the controls, the D-GalN group had severe hepatic tissue damage characterized by sinusoidal dilation, disrupted hepatic cord arrangement, and extensive inflammatory cell infiltration. Following ADSC transplantation, hepatic tissue damage was significantly improved, with reduced inflammatory cell infiltration and restored sinusoidal structure and hepatic cord arrangement (Figure 4).

### 2.5. ADSC Transplantation Improves Liver Tissue Fibrosis and Abnormal Glycogen Metabolism in ALI Rats

**Figure 5:**
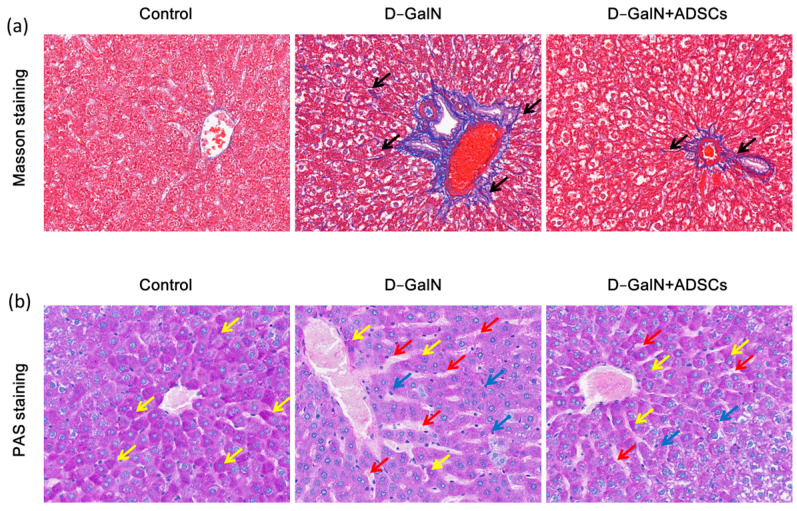

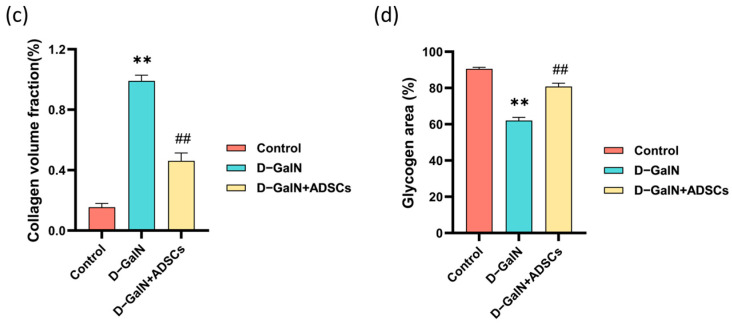
Masson trichrome staining and PAS staining. (**a**) Masson trichrome staining. Black arrows: collagen deposition. (**b**) PAS staining. Yellow arrows: glycogen deposition; blue arrows: hepatocyte necrosis; red arrows: sinusoidal dilatation. Scale bar = 100 μm. (**c**) Quantitative analysis of fibrotic area. (**d**) Quantitative analysis of glycogen area. Mean ± SD. ** *p* < 0.01 vs. control group; ^##^ *p* < 0.01 vs. D-GalN group. (*n* = 3).

Masson trichrome staining revealed diffuse collagen fiber deposition and structural disruption in liver tissue of the D-GalN group versus the controls, whereas ADSC transplantation significantly reduced fibrosis severity (Figure 5a,c). PAS staining revealed markedly reduced glycogen content (attenuated PAS staining) in hepatocytes of the D-GalN group, accompanied by lesions such as sinusoidal dilatation. Following ADSC transplantation, glycogen deposition increased and lesions diminished (Figure 5b,d). These findings indicate that ADSC transplantation effectively improves fibrosis and glycogen metabolism disorders.

### 2.6. ADSC Transplantation Improves Liver Function in ALI Rats

**Figure 6:**
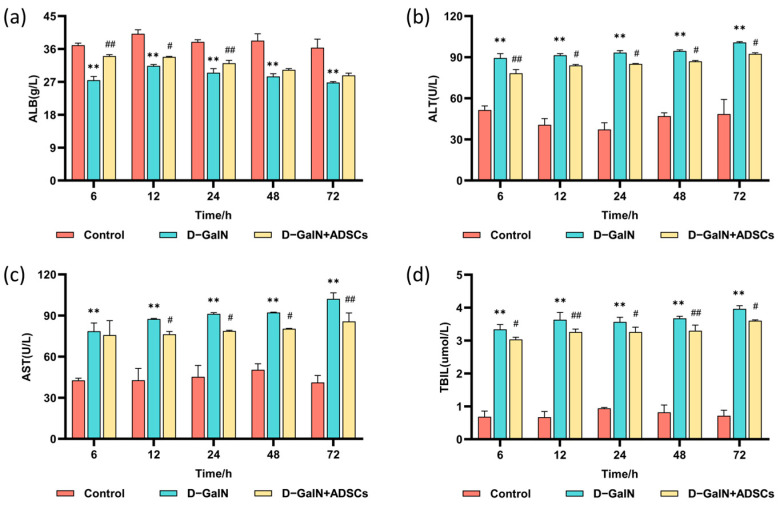
Serum liver function indicator levels. (**a**) ALB. (**b**) ALT. (**c**) AST. (**d**) TBIL. Mean ± SD. ** *p* < 0.01 vs. control group; ^#^ *p* < 0.05, ^##^ *p* < 0.01 vs. D-GalN group. (*n* = 3).

In comparison to the D-GalN group, ADSC transplantation markedly increased serum ALB levels and markedly decreased ALT, AST, and TBIL levels (Figure 6). Findings indicate that ADSC transplantation effectively improves liver function in ALI rats.

### 2.7. ADSC Transplantation Alleviates Inflammation in ALI Rats

**Figure 7:**
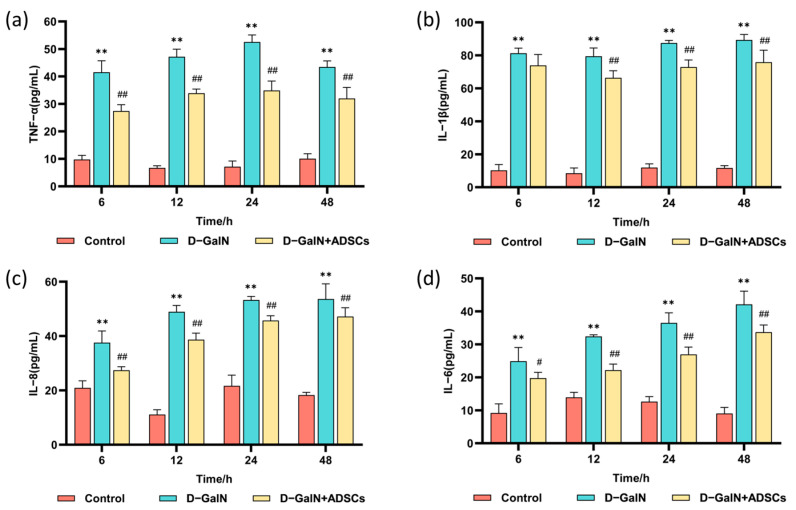
Serum inflammatory cytokine levels. (**a**) TNF-α. (**b**) IL-1β. (**c**) IL-8. (**d**) IL-6. Mean ± SD. ** *p* < 0.01 vs. control group; ^#^ *p* < 0.05, ^##^ *p* < 0.01 vs. D-GalN group. (*n* = 3).

A marked increase in serum TNF-α, IL-1β, IL-6, and IL-8 was observed in the D-GalN group relative to controls (*p* < 0.01). Following ADSC transplantation, levels of these proinflammatory factors were markedly reduced (*p* < 0.01; Figure 7). The results demonstrate that ADSC transplantation effectively attenuates the inflammatory response in ALI rats.

### 2.8. ADSC Transplantation Reduces Oxidative Stress-Related Markers in ALI Rats

**Figure 8:**
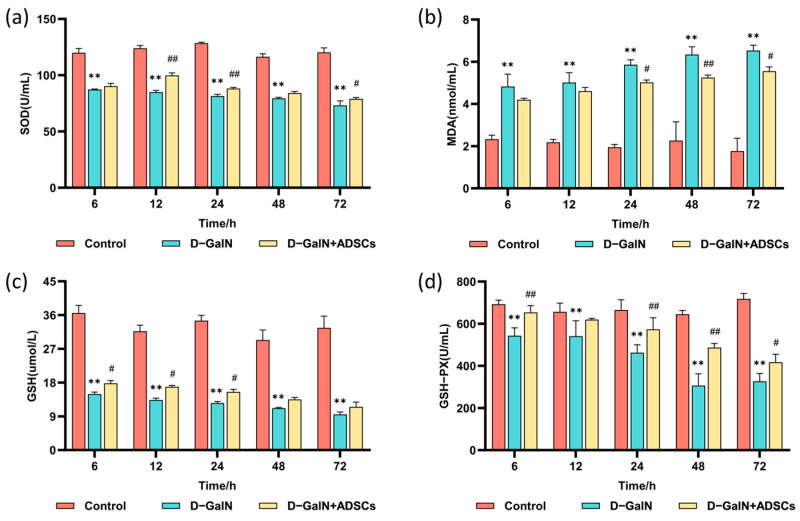
Serum oxidative stress markers. (**a**) SOD. (**b**) MDA. (**c**) GSH. (**d**) GSH-PX. Mean ± SD. ** *p* < 0.01 vs. control group; ^#^ *p* < 0.05, ^##^ *p* < 0.01 vs. D-GalN group. (*n* = 3).

Relative to the control group, serum antioxidant markers SOD, GSH, and GSH-PX activity were markedly reduced (*p* < 0.01) in the D-GalN group rats, while the level of lipid peroxidation product MDA (*p* < 0.01) was significantly elevated. ADSC transplantation effectively reversed this trend, significantly enhancing SOD, GSH, and GSH-PX activity while reducing MDA levels (Figure 8). These findings indicate that ADSC transplantation alleviates oxidative stress in ALI rats by enhancing antioxidant capacity and mitigating oxidative damage.

### 2.9. ADSC Transplantation Promotes Hepatocyte Regeneration in ALI Rats

**Figure 9:**
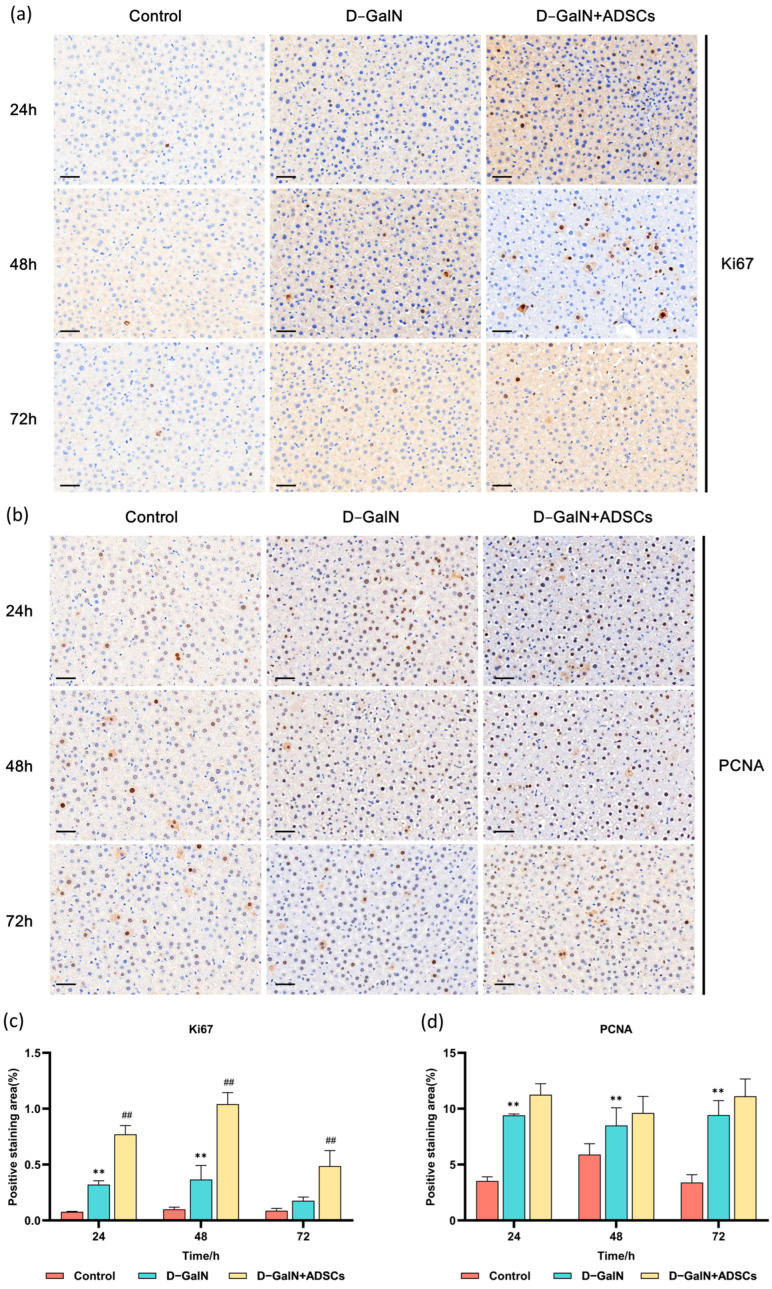
Immunohistochemical staining results for Ki67 and PCNA in liver tissue. (**a**) Representative staining images for Ki67, (**b**) PCNA (Scale bar = 100 μm). Quantitative analysis of (**c**) Ki67 and (**d**) PCNA positive expression. Mean ± SD. ** *p* < 0.01 vs. control group; ^##^ *p* < 0.01 vs. D-GalN group. (*n* = 3).

Immunohistochemical results revealed that both Ki67 and PCNA expression were significantly elevated in the D-GalN group (*p* < 0.01). Following ADSC transplantation, Ki67-positive expression was further significantly enhanced (*p* < 0.01); although PCNA expression showed an upward trend, the difference was not statistically significant (Figure 9). These findings indicate that ADSC transplantation promotes hepatocyte regeneration in rats with ALI.

### 2.10. ADSCs Downregulate TLR4/MyD88/NF-κB Pathway-Related Protein Expression in ALI Rats

**Figure 10:**
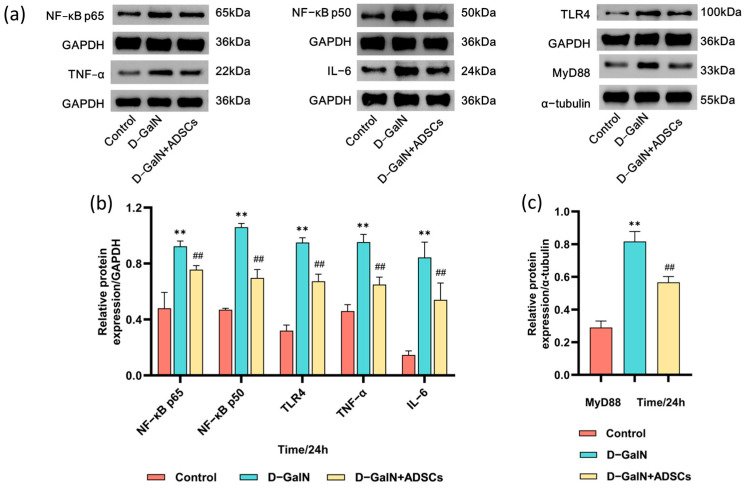
Western blot analysis of proteins associated with the TLR4/MyD88/NF-κB axis. (**a**) Band patterns of target proteins. (**b**,**c**) Quantitative analysis of relative expression levels of target proteins (Mean ± SD). Internal control notes: GAPDH was used as the internal control for TLR4, NF-κB p65/p50, TNF-α, and IL-6; α-tubulin was used for MyD88. ** *p* < 0.01 vs. control group; ^##^ *p* < 0.01 vs. D-GalN group. (*n* = 3).

Western blot analysis showed that D-GalN markedly upregulated hepatic levels of TLR4, MyD88, NF-κB p65, NF-κB p50, TNF-α, and IL-6 in ALI rats compared to the control group (*p* < 0.01). ADSC transplantation significantly reversed this trend, effectively reducing the expression of these key proteins (*p* < 0.01; Figure 10 and Figure S1). This indicates that ADSCs exert anti-inflammatory effects by suppressing the TLR4/MyD88/NF-κB axis.

### 2.11. Effects of ADSC Transplantation on mRNA Expression of Key Pathological Process-Related Genes in Liver Tissue of ALI Rats

**Figure 11:**
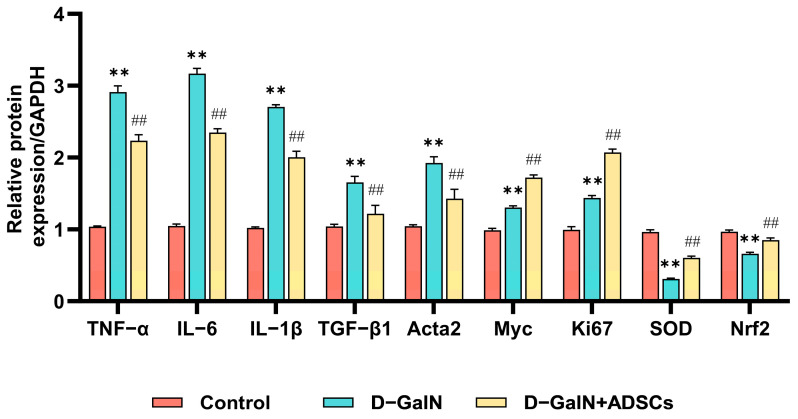
mRNA expression of key genes in liver tissue. qPCR was performed to quantify the expression of TNF-α, IL-6, IL-1β, TGF-β1, Acta2, Myc, Ki67, SOD, and Nrf2. Mean ± SD. ** *p* < 0.01 vs. control group; ^##^ *p* < 0.01 vs. D-GalN group. (*n* = 3).

Compared with the control group, proinflammatory factors (TNF-α, IL-6, IL-1β), pro-fibrotic genes (TGF-β1, Acta2), the proto-oncogene Myc, and the cell proliferation marker Ki67 were significantly upregulated in the liver tissue of D-GalN IL-1β, pro-fibrotic genes (TGF-β1, Acta2), the oncogene Myc, and the cell proliferation marker Ki67 were markedly enhanced (*p* < 0.01), while antioxidant-related genes (SOD, Nrf2) were markedly reduced (*p* < 0.01). Following ADSC transplantation, relative to the D-GalN group, the treatment group exhibited considerably reduced expression of proinflammatory factors and pro-fibrotic genes (*p* < 0.01), while Ki67, SOD, and Nrf2 expression was markedly increased (*p* < 0.01; Figure 11). This suggests that ADSC transplantation promotes hepatocyte regeneration and enhances tissue antioxidant stress resistance.

## 3. Discussion

We assessed the therapeutic efficacy of Luopan Mountain pig ADSCs against D-GalN-induced ALI in a rat model. The results indicated that ADSC transplantation significantly repaired and protected against liver injury by suppressing inflammatory responses, alleviating oxidative stress, promoting hepatocyte regeneration, and inhibiting collagen fiber deposition. Further investigations suggested that these protective effects may involve suppression of the TLR4/MyD88/NF-κB pathway.

The etiology of ALI is complex and multifactorial, with common triggers including drug toxicity (e.g., acetaminophen), exogenous toxins (e.g., aflatoxin B_1_), and pathogen infections (e.g., leptospirosis) [40,41]. These pathogenic factors typically act through core mechanisms such as directly disrupting hepatocyte membrane stability, inducing mitochondrial dysfunction, and activating oxidative stress and inflammatory signaling pathways, ultimately leading to massive hepatocyte apoptosis or necrosis [42]. Correspondingly, its typical pathological manifestations encompass hepatocyte injury, inflammatory cell infiltration, steatosis, and early fibrosis formation [43]. Currently, clinical management of ALI remains primarily supportive and symptomatic, lacking specific therapies targeting these multifaceted pathological pathways. This underscores the unique advantage of ADSC therapy as a comprehensive treatment strategy.

The detection of liver function markers serves as a key basis for evaluating liver injury and repair. This study successfully established a D-GalN-induced ALI model, characterized by significantly elevated serum ALT, AST, and TBIL, along with decreased ALB. Following autologous ADSC transplantation, these indicators showed marked improvement. This improvement trend is consistent with research reports using ADSCs from other species [44]. For instance, in a canine CCl_4_-induced acute liver injury model, canine ADSCs similarly significantly reduced serum AST and ALT levels [45]; in sodium diclofenac-induced rat liver injury, rat ADSCs also effectively improved ALT, AST, and ALB indicators [46]. Despite differing species origins, the beneficial effects of ADSCs in improving liver function markers are widely observed. At the mechanistic level, this study further confirmed that ADSCs effectively reversed D-GalN-induced oxidative stress by elevating SOD, GSH-PX, and GSH levels while reducing MDA content. This finding aligns with the antioxidant effects of MSCs reported in the literature [47,48], suggesting that ADSCs may enhance the body’s antioxidant defense system through multiple pathways. These include boosting free radical scavenging capacity [49], restoring antioxidant enzyme function [50,51], and inhibiting ROS production [52], thereby mitigating oxidative stress damage to hepatocytes. This effect was also validated in acute liver injury induced by rabbit RHDV2 virus, where treatment with rabbit ADSCs was accompanied by a reduction in oxidative stress [53].

MSCs have demonstrated clear therapeutic efficacy in the intervention of liver fibrosis. Research has shown that in CCl_4_-induced liver injury models, MSC transplantation significantly reduces collagen deposition in liver tissue. This effect can be visually observed through Masson staining, revealing markedly decreased collagen content and significantly reduced fibrosis severity in treated liver tissue [54]. Further mechanistic studies indicate that MSCs also reduce fibrosis at its source by inhibiting hepatic stellate cell activation (manifested as downregulation of α-SMA expression) [55]. In this study, following D-GalN-induced ALI, rat liver tissue exhibited severe fibrotic proliferation, with lesions primarily concentrated in the central veins and portal areas (with more pronounced damage in the portal areas). Fibrous septa even partitioned normal liver tissue into pseudo-lobules. However, after ADSC transplantation, the degree of liver fibrosis was markedly alleviated. with clearly discernible portal tract and central vein structures. This finding strongly aligns with existing MSCs’ anti-fibrotic studies in the liver, further confirming the inhibitory effect of ADSCs on the fibrotic process during liver injury repair.

Regarding the restoration of hepatic metabolic function, the regulatory effects of MSCs on glycogen synthesis and glucose metabolism have also been documented in multiple studies. For instance, in a model of ALF induced by 90% partial hepatectomy (PH), MSCs transplantation significantly elevated blood glucose levels and promoted hepatocyte regeneration [56]. In CCl_4_-induced liver fibrosis models, both MSCs and their conditioned medium (MSC-CM) promoted hepatic glycogen synthesis and storage, improved liver function markers (ALT, AST), and accelerated hepatocyte regeneration [57]. Another study using PAS staining analysis revealed significantly increased glycogen content in liver tissue after MSC treatment, suggesting effective restoration of hepatocyte metabolic function [58]. Consistent with these findings, PAS staining in this study revealed markedly reduced glycogen deposition in the liver tissue of D-GalN-treated rats. Following ADSC transplantation, glycogen deposition partially recovered, indicating that ADSCs may facilitate liver tissue functional repair by regulating glycogen metabolism. Furthermore, this study found that the expression of hepatocyte proliferation markers Ki67 and PCNA was significantly upregulated after ADSC transplantation. This aligns with the findings of Ding et al. [56], suggesting that ADSCs possess the capacity to promote hepatocyte regeneration.

In D-GalN-induced acute liver injury, the TLR4/MyD88/NF-κB signaling pathway plays a pivotal role by regulating inflammatory responses and apoptosis, thereby influencing the severity of liver injury and the repair process [59]. As a major member of the Toll-like receptor family, TLR4 recognizes endogenous DAMPs (such as HMGB1) within LPS and initiates downstream signaling pathways [60]. Research indicates that UMSCs suppress NF-κB activation by downregulating MyD88 expression, thereby reducing proinflammatory factor release and improving mouse survival rates [61]. Our findings demonstrate that D-GalN-induced ALI significantly elevated TNF-α, IL-6, IL-1β, and IL-8 expression, which improved after ADSC transplantation. Concurrently, HE staining revealed substantial inflammatory cell infiltration in the injured liver tissue. Following ADSC transplantation, these injury markers also showed improvement, consistent with the findings of Piao et al. [62]. This confirms that ADSCs effectively suppress proinflammatory cytokine release and mitigate D-GalN-induced liver injury. Furthermore, Western blot analysis in this study demonstrated that after D-GalN-induced liver injury, MyD88, IL-6, and TLR4 were significantly elevated in the D-GalN-induced liver tissue. Following ADSC transplantation, this signaling pathway was suppressed, and inflammatory mediator expression was reduced, consistent with previous studies [63,64]. These findings further confirm that ADSCs effectively suppress liver injury while protecting and repairing hepatic function. This protective effect likely occurs through suppression of the TLR4/MyD88/NF-κB axis.

## 4. Materials and Methods

### 4.1. Preparation and Characterization of ADSCs

#### 4.1.1. In Vitro Isolation and Culture of ADSCs

Under aseptic conditions, the adipose tissue was harvested from Luopan Mountain pigs. After rinsing with PBS, the tissue was minced into approximately 1 mm^3^ pieces. A solution of 0.2% type II collagenase was added and digested at 37 °C for 30 min. Digestion was terminated with H-DMEM medium containing 13% FBS. Samples were spun at 1200 r/min for 6 min. The collected cell pellet was resuspended and seeded for culture in a 37.5 °C, 5% CO_2_ incubator. When cell confluence reached over 90%, cells were cryopreserved at 1 × 10^6^ cells/mL for future use.

#### 4.1.2. Immunofluorescence Identification of Surface Markers on ADSCs

P4-passaged ADSCs were seeded into confocal culture dishes. When confluence exceeded 90%, the culture medium was removed and fixed for 30 min. Cells were permeabilized with 0.25% Triton X-100 for 20 min. Subsequently, the cells were blocked with goat serum for 30 min. The blocking solution was discarded, and rabbit-derived primary antibodies against CD44, CD73, CD90, and CD105 (Bioss, Beijing, China) were added and kept at 4 °C for 24 h. After washing with PBS, we added FITC-labeled goat anti-rabbit secondary antibody (Bioss, Beijing, China) and incubated the samples in the dark for 1 h. To label the nuclei, the samples were subjected to a 15 min DAPI staining. We acquired images on a laser confocal microscope.

#### 4.1.3. Validation of Multipotent Differentiation Potential of ADSCs

P4-generation ADSCs with a confluence of 60% or higher were cultured with adipogenic, osteogenic, and chondrogenic induction media (for 14 days, 21 days, and 21 days, respectively). Following morphological changes indicative of induced differentiation, cells were subjected to Oil Red O (adipogenic), Alizarin Red (osteogenic), and Alizarin Blue (chondrogenic) to assess differentiation status. Induction medium formulations are provided in Supplementary Material (Table S1).

### 4.2. Experimental Animals and ALI Model Establishment

We randomly assigned forty-five eight-week-old SD rats (190–210 g, equal sexes) to three groups (*n* = 15): the control rats were injected i.p. with saline; model rats received 600 mg/kg D-GalN i.p.; and the D-GalN + ADSCs group received the same D-GalN dose i.p., followed by 2 × 10^6^ ADSCs via tail vein injection. All animal experiments were authorized by the Animal Ethics Committee of the Institute of Animal Science, Chinese Academy of Agricultural Sciences (IAS2025-128). All experimental procedures adhered to the ARRIVE guidelines. We collected blood and liver tissues from three anesthetized rats per group at each time point (6, 12, 24, 48, and 72 h) following ADSC transplantation.

### 4.3. Homeotaxis Assay for ADSCs

ADSCs were labelled with CM-Dil fluorescent dye (YEASEN, Shanghai, China) by incubating at 37 °C for 30 min. Two hours after D-GalN-induced acute liver injury, CM-Dil-labeled ADSCs (2 × 10^6^ cells/mL, dissolved in PBS) were administered via tail vein injection. Rats were euthanized 24 h post-injection, and liver tissue was harvested. Fluorescent microscopy was used to observe the localization and distribution of labeled ADSCs within the liver tissue.

### 4.4. Blood Biochemical Indicator Testing

Serum ALB, AST, TBIL, and ALT levels were determined with an automated chemistry analyzer (Mindray, Shenzhen, China).

### 4.5. Serum Cytokine Testing

Serum levels of TNF-α, IL-8, IL-6, and IL-1β were detected using an ELISA kit (JONLNBIO, Jinan, China).

### 4.6. Detection of Oxidative Stress Markers in Liver Tissue

ELISA (BIOBASE, Jinan, China) was used to detect MDA and GSH levels, as well as SOD and GSH-PX activities in liver tissue.

### 4.7. Hepatic Tissue Pathological Analysis

Following fixing in 4% paraformaldehyde, liver tissues were paraffin-embedded and sectioned at 5 μm. These sections underwent HE, Masson’s trichrome, and PAS staining to assess pathological changes. Observations were made under an optical microscope, and images were captured.

### 4.8. Immunohistochemical Detection

Following deparaffinization and rehydration, the liver tissue sections were blocked with goat serum to minimize nonspecific binding. We incubated the sections with primary antibodies (PCNA, 1:2000, PTC; Ki67, 1:500, Huilan Bio, Guangzhou, China) at 4 °C overnight. Membranes were incubated with an HRP-conjugated goat anti-rabbit secondary antibody (1:500, Pinofei Bio, Wuhan, China) at 37 °C for 1 h. After DAB development and nuclear hematoxylin counterstaining, slides were dehydrated and mounted.

### 4.9. Western Blotting

Total liver protein was isolated using RIPA lysis buffer (Pinnovo Bio, Shenzhen, China). After separation by SDS-PAGE, proteins were blotted onto PVDF membranes (Thermo Fisher Scientific, Waltham, MA, USA). Following primary antibody incubation (4 °C, overnight), membranes were then probed with the HRP-conjugated goat anti-rabbit secondary antibody (1:2000, ab97051, Abcam, Cambridge, UK) for 1 h at room temperature. Revelation was performed using ECL reagents (Pinnacle Bio, Changzhou, China), and band intensity was analyzed using ImageJ 1.52i software. Primary antibodies used included the following: TLR4 (1:8000, 66350-1-IG, Proteintech, Wuhan, China), MyD88 (1:3000, 67969-1-IG, Proteintech, Wuhan, China), NF-κB p65 (1:5000, CY5034, Abways, Shanghai, China), TNF-α (1:4000, 60291-1-IG, Proteintech, Wuhan, China), IL-6 (1:2000, R1412-2, Huabio, Hangzhou, China), and NF-κB p50 (1:2000, CY5040, Abways, Shanghai, China).

### 4.10. qPCR

The mRNA levels of TNF-α, IL-6, IL-1β, TGF-β1, Acta2, Myc, Ki67, SOD, and Nrf2 were quantified via qPCR using SYBR Green MasterMix (A25742, Thermo Fisher Scientific, Waltham, MA, USA). Relative gene expression was calculated using the 2^−ΔΔCt^ method after normalization to GAPDH. Primer sequences are detailed in Supplementary Table S2.

### 4.11. Statistical Analysis

Statistical analysis was performed with GraphPad Prism (v10.1.2), with all data shown as mean ± SD. Multiple group comparisons were analyzed by one-way ANOVA, and bivariate analysis by two-way ANOVA. Statistical significance was set at *p* < 0.05, and Tukey’s test was conducted for post hoc analysis.

## 5. Conclusions

This study demonstrates for the first time that transplantation of ADSCs isolated from Luopan Mountain pigs exerts significant therapeutic effects in D-GalN-induced ALI rats. The core mechanism involves suppressing the excessive triggering of the TLR4/MyD88/NF-κB signaling pathway, thereby facilitating liver injury repair through multi-level synergistic actions. On one hand, it directly downregulates the release of proinflammatory factors (TNF-α, IL-1β, IL-6, IL-8) by inhibiting this pathway, thereby alleviating inflammatory responses. On the other hand, it promotes liver function recovery and tissue regeneration through multiple effects: enhancing antioxidant capacity (increasing SOD and GSH-PX activity while reducing MDA levels), promoting hepatocyte regeneration (upregulating Ki67 expression), and inhibiting liver fibrosis (downregulating TGF-β1/Acta2 expression). This study not only elucidates the molecular mechanisms underlying the synergistic repair of ALI by Luopan Mountain pig ADSCs, filling a gap in related research, but also provides robust experimental evidence and potential strategies for cell therapy in acute liver injury.

## Data Availability

Data supporting the results of this study are available from the corresponding author upon request.

## Supplementary Materials

The supporting information can be downloaded at: https://www.mdpi.com/article/10.3390/ijms262411798/s1.

## Abbreviations

The following abbreviations are used in this manuscript:

MSCs	Mesenchymal stem cells
ADSCs	Adipose-derived mesenchymal stem cells
D-GalN	D-galactosamine
ALI	Acute liver injury
