# Supplementary material for: Adipose-Derived Mesenchymal Stem Cells Improve Acute Liver Injury: A Mechanistic Study Based on the TLR4/MyD88/NF-κB Pathway
Source: Int J Mol Sci. 2025 Dec 6;26(24):11798. doi: 10.3390/ijms262411798 (PMC12732611; doi:10.3390/ijms262411798)
Supplement: Supplementary file 1 [file ijms-26-11798-s001.zip › Table S1.pdf]

Table S1. Formulations of Induction Media

Osteoblasts	Basal medium:High-glucose DMEM<br>Supplements:10% FBS (fetal bovine serum)+1% penicillin-streptomycin+<br>0.5 mM 3-isobutyl-1-methylxanthine (IBMX)+10 μg/mL insulin+<br>1 μM dexamethasone+200 μM indomethacin
Adipocytes	Basal medium: High-glucose DMEM<br>Supplements:2.5% FBS+1% penicillin-streptomycin+0.1 μM dexamethasone+<br>10 mM β-glycerophosphate sodium+50 μg/mL ascorbic acid
Chondrocytes	Basal medium: High-glucose DMEM<br>Supplements:2.5% FBS+1% penicillin-streptomycin+1% insulin+50 μg/mL<br>L-proline+0.1 μM dexamethasone+0.9 mM sodium pyruvate+<br>10 ng/mL transforming growth factor-beta 3 (TGF-β3)+50 μg/mL ascorbic acid<br>(Vc)+1% insulin-transferrin-selenium (ITS)
