# Supplementary material for: Adipose-Derived Mesenchymal Stem Cells Improve Acute Liver Injury: A Mechanistic Study Based on the TLR4/MyD88/NF-κB Pathway
Source: Int J Mol Sci. 2025 Dec 6;26(24):11798. doi: 10.3390/ijms262411798 (PMC12732611; doi:10.3390/ijms262411798)
Supplement: Supplementary file 1 [file ijms-26-11798-s001.zip › Table S2.pdf]

Table S2. Primers for quantitative real-time PCR analysis.

Gene	Primer
GAPDH	ACAGCAACAGGGTGGTGGAC
	TTTGAGGGTGCAGCGAACTT
TNF-α	CAGACCCTCACACTCAGATCAT
	AGATAAGGTACAGCCCATCTGC
IL-6	CTCTCCGCAAGAGACTTCCA
	TCTCCTCTCCGGACTTGTGAA
IL-1β	CCTGTGTGATGAAAGACGGC
	TATGTCCCGACCATTGCTGT
TGF-β1	CCCTACATTTGGAGCCTGGA
	CGCACGATCATGTTGGACAA
Acta2	GGATCAGCGCCTTCAGTTCT
	CAGGGCTAGAAGGGTAGCAC
Myc	CTCGGTGCAGCCCTATTTCA
	TAGCGACCGCAACATAGGAC
Ki67	CACCAGAGCCAATAGATACTTC
	GTGTCCAATTTCCGCTTTAC
SOD	AGCTTGTCAGGTGTGGAACC
	GCGTGTCGCCTATCTTCTCA
Nrf2	TGACTCTGACTCCGGCATTT
	CCCCAGAAGAATGTGTTGGC
