# Supplementary figures and images for: Adipose-Derived Mesenchymal Stem Cells Improve Acute Liver Injury: A Mechanistic Study Based on the TLR4/MyD88/NF-κB Pathway
Source: Int J Mol Sci. 2025 Dec 6;26(24):11798. doi: 10.3390/ijms262411798 (PMC12732611; doi:10.3390/ijms262411798)

Figure S1 Original Western blot

(1)

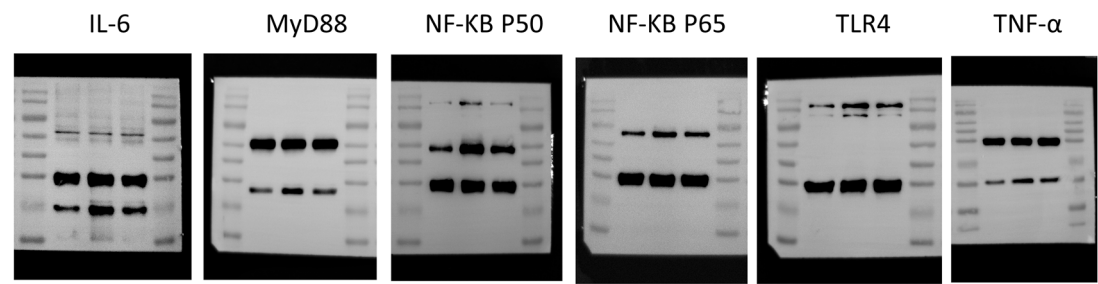

(2)

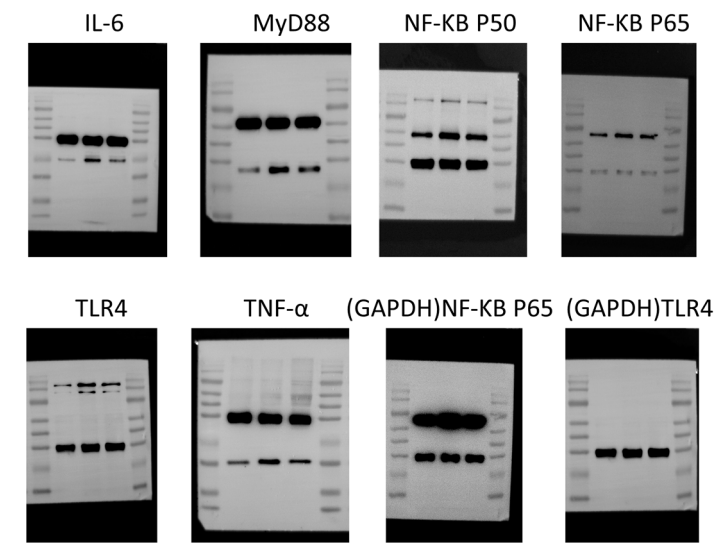

(3)

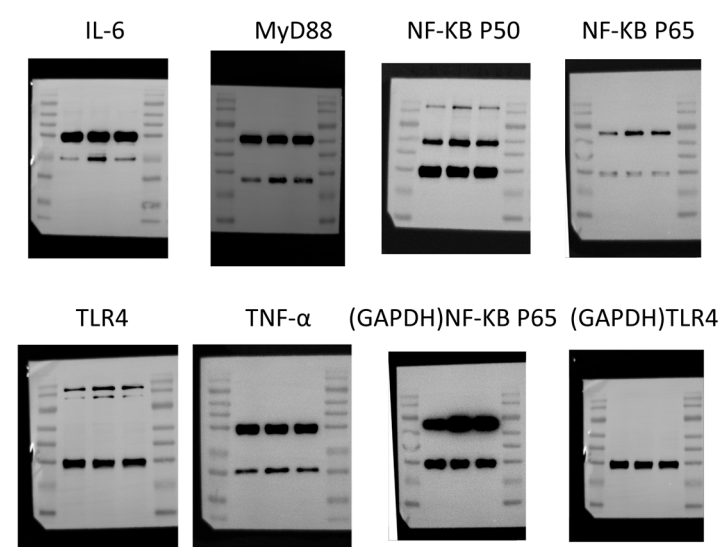

Supplement: Supplementary file 1 [file ijms-26-11798-s001.zip › Figure S1.pdf]
